# Supplementary material for: Unilateral loss of recql4 function in Xenopus laevis tadpoles leads to ipsilateral ablation of the forelimb, hypoplastic Meckel's cartilage, and vascular defects
Source: G3 (Bethesda). 2025 Aug 16;15(10):jkaf179. doi: 10.1093/g3journal/jkaf179 (PMC12506664; doi:10.1093/g3journal/jkaf179)
Supplement: jkaf179_Supplementary_Data [file jkaf179_supplementary_data.zip › Supplementary_Figure_1_G3-2025-406107.docx]

| 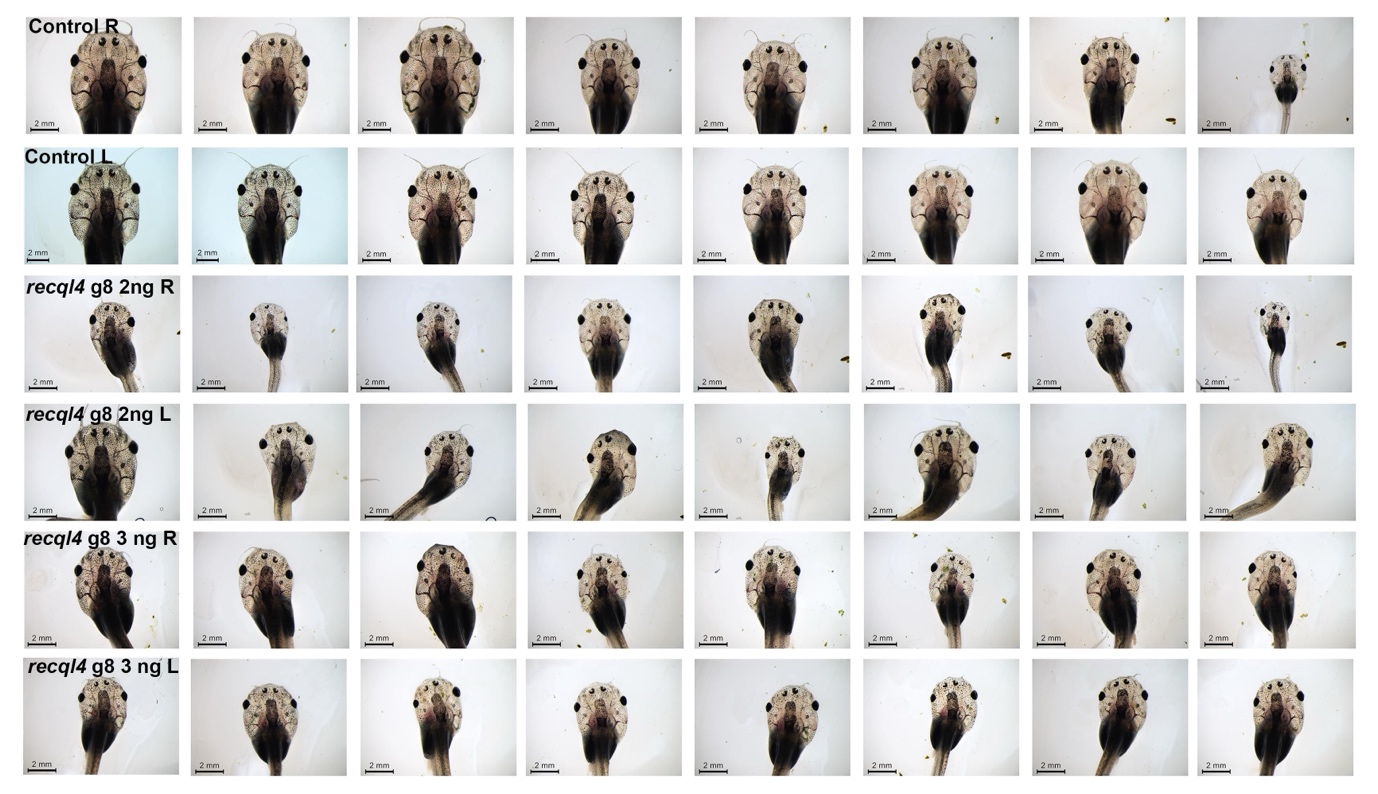 | |
| --- | --- |
| 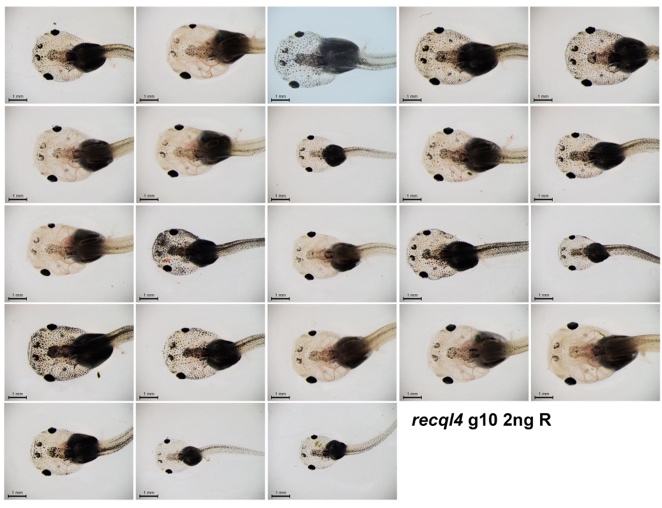 | 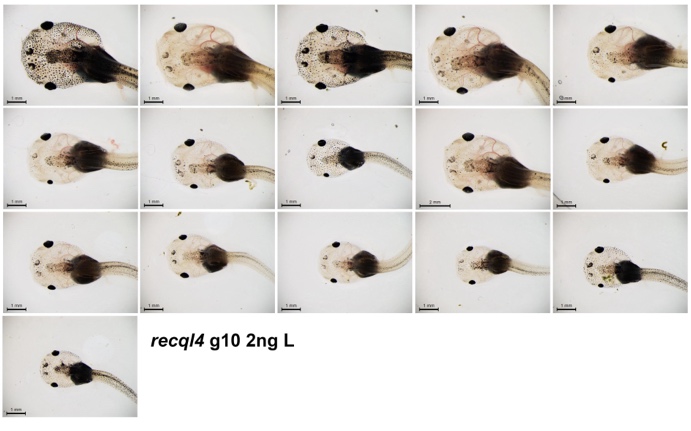 |
| **Supplementary Figure 1: Examples of *recql4* CRISPR half tadpoles at 3 weeks of development (Figure 2E,F)** Top: first tadpole batch, controls are injected with just GFP mRNA, CRISPants using sgRNA8, scale bars 2 mm. Dorsal view of head and trunk with anterior uppermost. Bottom second tadpole batch using sgRNA10, dorsal view of head and trunk with head to left, scale bars 1 mm. L, injected on Left side, R, injected on right side. | |
