# Supplementary material for: Unilateral loss of recql4 function in Xenopus laevis tadpoles leads to ipsilateral ablation of the forelimb, hypoplastic Meckel's cartilage, and vascular defects
Source: G3 (Bethesda). 2025 Aug 16;15(10):jkaf179. doi: 10.1093/g3journal/jkaf179 (PMC12506664; doi:10.1093/g3journal/jkaf179)
Supplement: jkaf179_Supplementary_Data [file jkaf179_supplementary_data.zip › Supplementary_Figure_2_G3-2025-406107.docx]

| 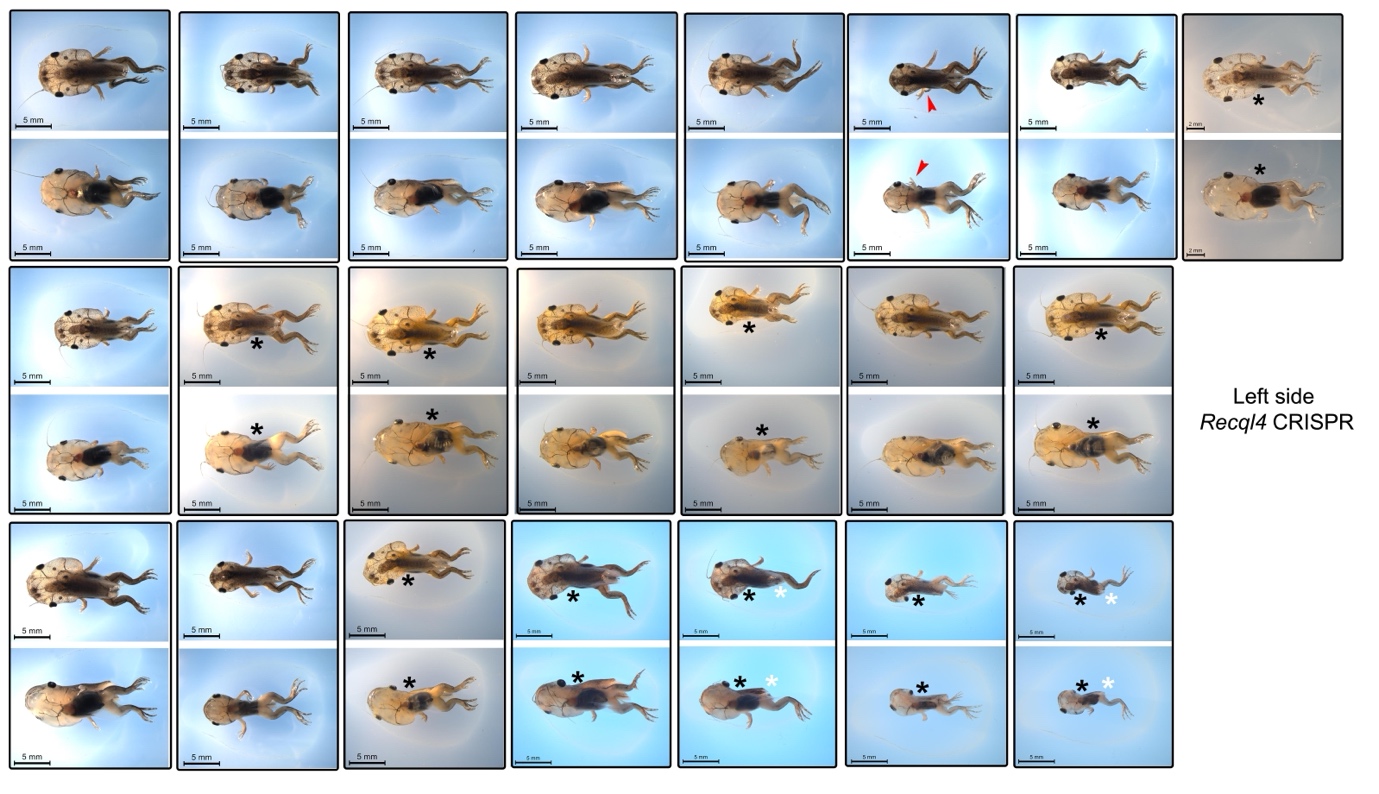 |
| --- |
| 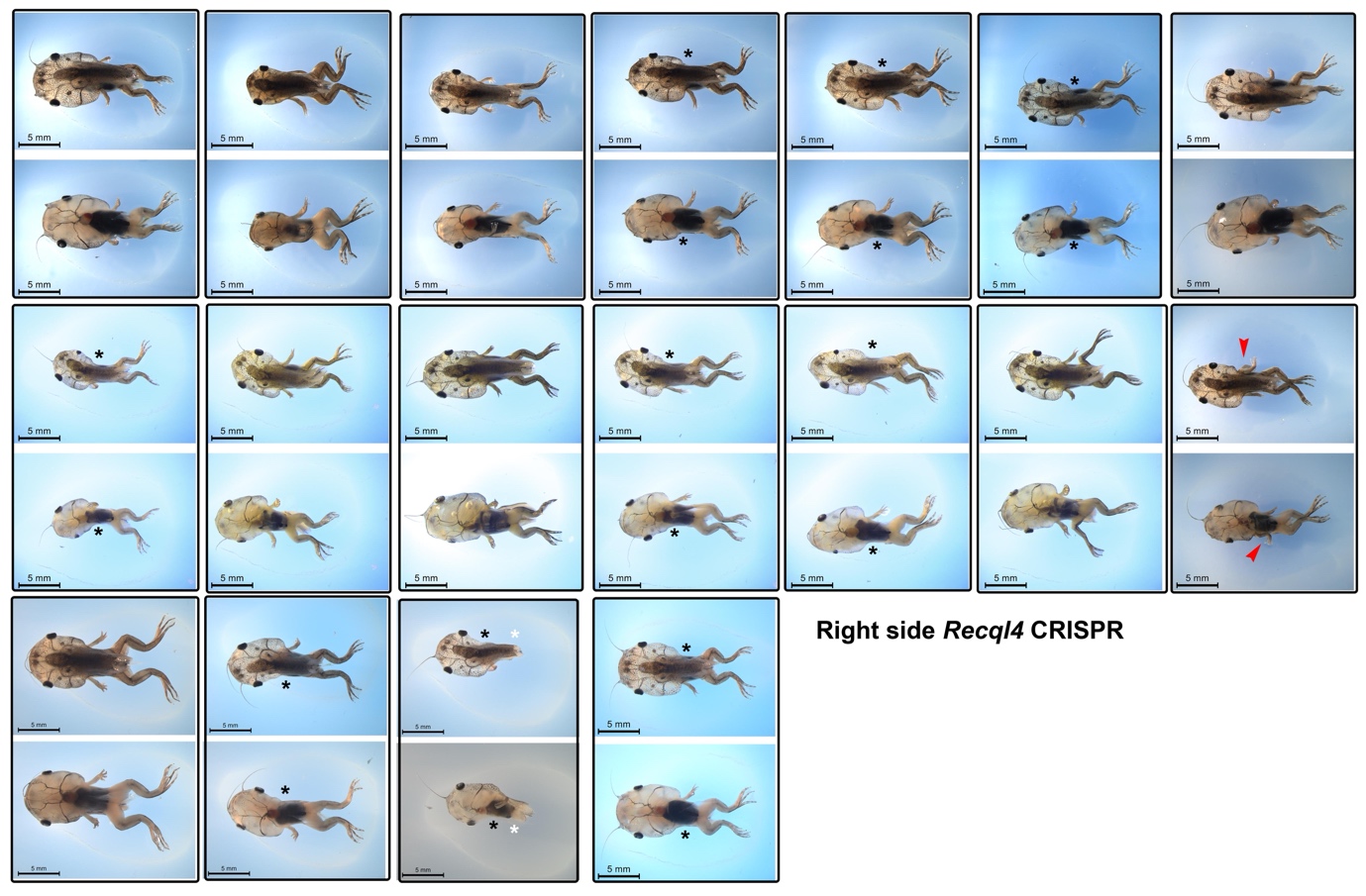 |
| **Supplementary Figure 2 Stage 58 half CRISPR *recql4* sgRNA8 tadpoles (Figures 3, 4).** Dorsal and ventral views for each animal are boxed, tails have been removed. Top, Left side CRISPants, Bottom, right side CRISPants, red arrowheads indicate ectopic limbs, black asterisk indicates a missing forelimb, white asterisk indicates a missing hindlimb. Scale bars 5 mm. |
