# Supplementary material for: Unilateral loss of recql4 function in Xenopus laevis tadpoles leads to ipsilateral ablation of the forelimb, hypoplastic Meckel's cartilage, and vascular defects
Source: G3 (Bethesda). 2025 Aug 16;15(10):jkaf179. doi: 10.1093/g3journal/jkaf179 (PMC12506664; doi:10.1093/g3journal/jkaf179)
Supplement: jkaf179_Supplementary_Data [file jkaf179_supplementary_data.zip › Supplementary_Figure_3_G3-2025-406107.docx]

| 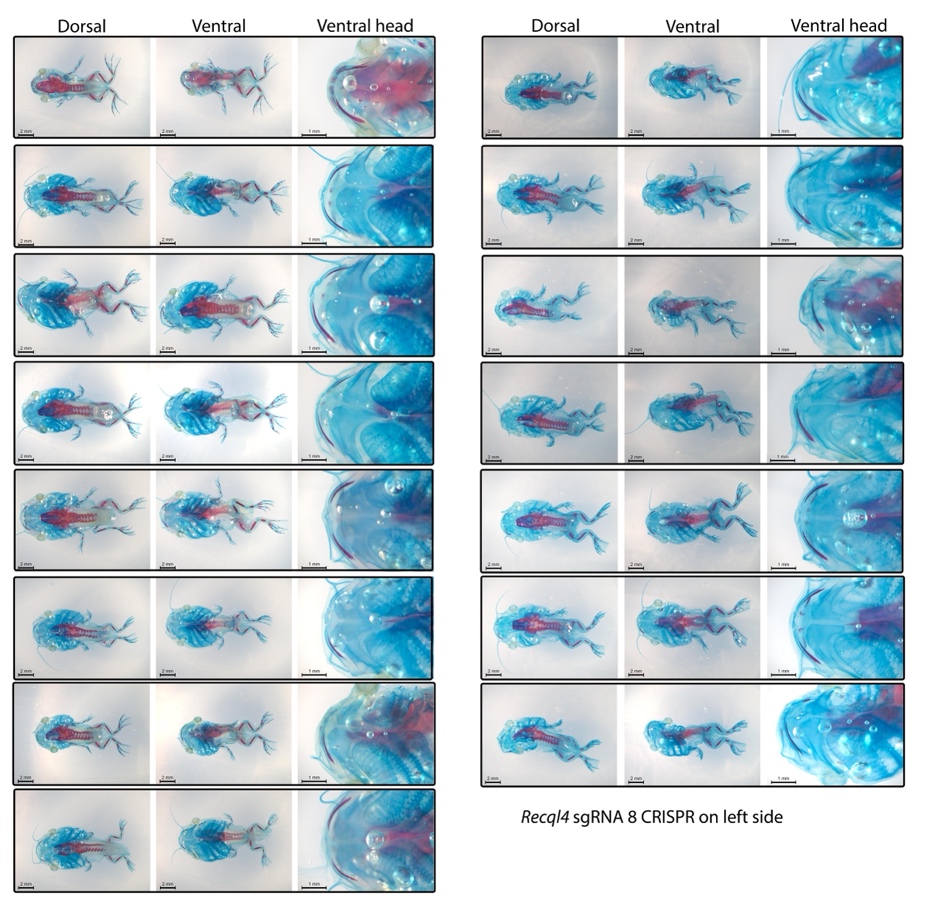 |
| --- |
| 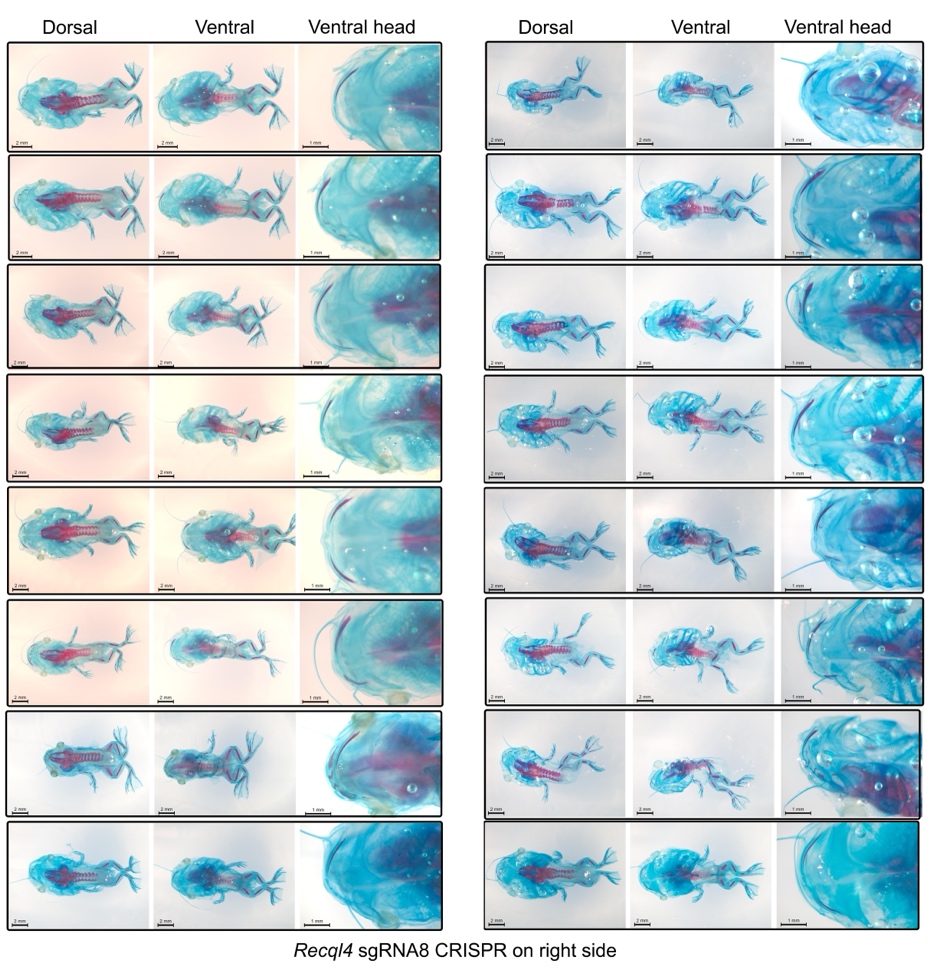 |
| **Supplementary Figure 3: Cartilage (blue) and bone (red) staining of half recql4 CRISPants (Figures 3, 4)**. Individual tadpoles are boxed, dorsal (left) and ventral (centre) showing whole stage 58 tadpole, right image is a zoom of the head cartilages from the ventral side. Top: left CRISPants, Bottom: right CRISPants. |
