# Supplementary material for: Unilateral loss of recql4 function in Xenopus laevis tadpoles leads to ipsilateral ablation of the forelimb, hypoplastic Meckel's cartilage, and vascular defects
Source: G3 (Bethesda). 2025 Aug 16;15(10):jkaf179. doi: 10.1093/g3journal/jkaf179 (PMC12506664; doi:10.1093/g3journal/jkaf179)
Supplement: jkaf179_Supplementary_Data [file jkaf179_supplementary_data.zip › Supplementary_Figure_4_G3-2025-406107.docx]

| 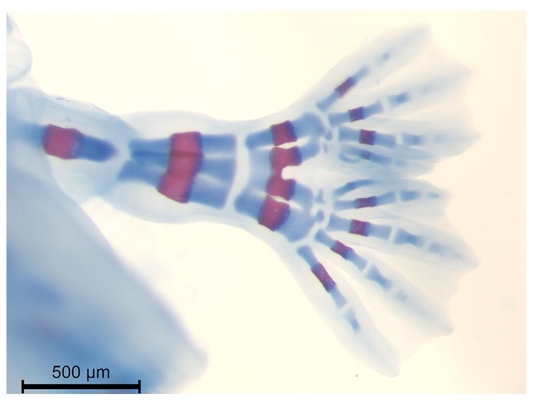 |
| --- |
| **Supplementary Figure 4: Ectopic hindlimb skeletal staining.** Individual from Figure 4C, stage NF55). There is a single femur, two fibulae and one central tibia, four tarsal bones and two complete hindfeet, with the two copies of digit 1 (hallux) adjacent. Viewed from the dorsal side |
